# 4058 Enhanced efficiency of large-scale clinical proteomic studies: when less is more

**DOI:** 10.1017/cts.2020.329

**Published:** 2020-07-29

**Authors:** Stefani Thomas, Betty Friedrich, Michael Schnaubelt, Daniel W. Chan, Hui Zhang, Ruedi Aebersold

**Affiliations:** 1 University of Minnesota; 2 Dept of Biology, Inst of Molec Systems Biology, ETH Zurich; 3 Johns Hopkins University; 4 ETH Zurich; University of Zurich

## Abstract

OBJECTIVES/GOALS: Large-scale clinical proteomic studies of cancer tissues often entail complex workflows and are resource-intensive. In this study we analyzed ovarian tumors using an emerging, high-throughput proteomic technology termed SWATH. We compared SWATH with the more widely used iTRAQ workflow based on robustness, complexity, ability to detect differential protein expression, and the elucidated biological information. METHODS/STUDY POPULATION: Proteomic measurements of 103 clinically-annotated high-grade serous ovarian cancer (HGSOC) tumors previously genomically characterized by The Cancer Genome Atlas were conducted using two orthogonal mass spectrometry-based proteomic methods: iTRAQ and SWATH. The analytical differences between the two methods were compared with respect to relative protein abundances. To assess the ability to classify the tumors into subtypes based on proteomic signatures, an unbiased molecular taxonomy of HGSOC was established using protein abundance data. The 1,599 proteins quantified in both datasets were classified based on z-score-transformed protein abundances, and the emergent protein modules were characterized using weighted gene-correlation network analysis and Reactome pathway enrichment. RESULTS/ANTICIPATED RESULTS: Despite the greater than two-fold difference in the analytical depth of each proteomic method, common differentially expressed proteins in enriched pathways associated with the HGSOC Mesenchymal subtype were identified by both methods. The stability of tumor subtype classification was sensitive to the number of analyzed samples, and the statistically stable subgroups were identified by the data from both methods. Additionally, the homologous recombination deficiency-associated enriched DNA repair and chromosome organization pathways were conserved in both data sets. DISCUSSION/SIGNIFICANCE OF IMPACT: SWATH is a robust proteomic method that can be used to elucidate cancer biology. The lower number of proteins detected by SWATH compared to iTRAQ is mitigated by its streamlined workflow, increased sample throughput, and reduced sample requirement. SWATH therefore presents novel opportunities to enhance the efficiency of clinical proteomic studies.

